# Protein Identification and Quantification Using Porous Silicon Arrays, Optical Measurements, and Machine Learning

**DOI:** 10.3390/bios13090879

**Published:** 2023-09-09

**Authors:** Simon J. Ward, Tengfei Cao, Xiang Zhou, Catie Chang, Sharon M. Weiss

**Affiliations:** 1 Department of Electrical and Computer Engineering, Vanderbilt University, Nashville, TN 37235, USA; simon.j.ward@vanderbilt.edu (S.J.W.);; 2 Interdisciplinary Material Science Program, Vanderbilt University, Nashville, TN 37235, USA; 3 Department of Chemistry, Vanderbilt University, Nashville, TN 37235, USA

**Keywords:** biosensing, porous silicon, sensor array, machine learning, dimensionality reduction, point-of-care, electronic noses, linear discriminant analysis, support vector machines, principal component analysis

## Abstract

We report a versatile platform based on an array of porous silicon (PSi) thin films that can identify analytes based on their physical and chemical properties without the use of specific capture agents. The ability of this system to reproducibly classify, quantify, and discriminate three proteins separately is demonstrated by probing the reflectance of PSi array elements with a unique combination of pore size and buffer pH, and by analyzing the optical signals using machine learning. Protein identification and discrimination are reported over a concentration range of two orders of magnitude. This work represents a significant first step towards a low-cost, simple, versatile, and robust sensor platform that is able to detect biomolecules without the added expense and limitations of using capture agents.

## 1. Introduction

The need to detect biological analytes for applications including medical diagnostics, environmental monitoring, and food safety, is typically met by a biosensor composed of two primary components: a capture agent (also sometimes referred to as a probe molecule or bioreceptor), which specifically binds to the desired target analyte, and a transducer, which converts the binding of the target species into a measurable optical, electrochemical, thermal, or microelectromechanical signal [1,2,3,4,5,6,7]. Examples of specific capture agent–target analyte ‘lock and key’ interactions are antibody–antigen interactions, enzyme–substrate interactions, peptide interactions, and oligonucleotide interactions [8,9,10]. However, despite their effectiveness and well-established use in many applications, the reliance on capture agents for analyte detection can bring about several challenges. The first is that a capture agent, by design, typically only binds to one species. If the aim is to identify and quantify multiple molecular constituents in a solution of unknown composition, multiple capture agents are required, which leads to larger, more complex, and expensive biosensors. Furthermore, if the appropriate capture agents are not present on a sensor, there is a possibility of hazardous species going undetected. In addition, many capture agents denature or degrade over time and/or in harsh environments, which limits the shelf life, ease of transportation, and types of locations where the sensor can be used [11,12,13]. Finally, when challenged with detecting a new target molecule of interest for which there is no existing capture agent, it can take significant time to develop an effective capture agent with sufficient specificity and affinity [14,15,16].

While there are molecular sensing approaches that do not require capture agents, they are not without their challenges as well. For example, spectroscopic techniques and many other protein detection assays typically require costly and bulky instrumentation, and their performance for analytes in complex media can often be limited [17]. Moreover, cost, ease and scalability of manufacture, and high sensitivity remain significant challenges for molecular imprinting approaches [18,19]. In addition, electronic noses or tongues typically face a trade-off between sensitivity and robustness, stability, complexity, and cost, especially when operating outside of controlled environments [20,21,22,23,24,25,26,27]. Different sensor platforms used in cross-reactive sensor arrays have their own inherent limitations. For example, colorimetric sensor arrays, which have been the subject of wide research interest, are limited in terms of their number of array elements due to the complexity of their manufacture, reproducibility, and printing quality; the minimum spot size of chemo-responsive dyes as a result of edge effects and a limited printing resolution; and their utility in detecting analytes in the aqueous phase, which poses additional significant challenges and often results in underwhelming detection limits (>1 μM) [21].

In this work, we report an initial demonstration showing that, under the appropriate preparation conditions, an array of porous silicon (PSi) sensors [28,29,30,31,32,33,34] has the potential to robustly classify, quantify, and discriminate a select number of molecular species without capture agents, based on their size, conformation, and surface charge. In particular, we demonstrate the detection of three proteins—bovine serum albumin (BSA), chicken ovalbumin (OVA), and avidin—separately, without the use of capture agents. These proteins, suspended in buffer solutions with varying pHs, are exposed to PSi films with different pore sizes. The proteins were chosen for this initial demonstration based on their overlapping combinations of isoelectric points and molecular weights, and because they are well characterized, which allows for the incorporation of prior knowledge into the machine learning analysis. The proteins can be discriminated at concentrations down to at least 300 nM, meeting clinically relevant detection limits for many applications [35,36,37] with 100% accuracy when the discrete set of possible concentrations is known, and 87.5% accuracy when classifying the protein type only for unseen concentrations. Given the large internal surface area and strong light matter interaction afforded by PSi, we anticipate that lower detection limits are achievable with further refinement of the platform and the inclusion of more degrees of freedom in the array element design and experimental testing conditions. The novel approach presented here for protein identification and quantification uniquely bridges the trade-off between the robustness and sensitivity of current sensor arrays by probing a wide reactivity space (encompassing more than purely hydrophobicity [20]) without requiring any surface treatments. Furthermore, PSi sensor arrays are cost effective, straightforward to fabricate, and easily scalable to high-volume manufacturing and large numbers of sensor array elements, to an extent which is not possible with most other platforms. This work opens the door to more advanced studies investigating the limits of using capture-agent-free PSi arrays for molecular identification and quantification in mixtures and other complex solutions.

## 2. Materials and Methods

### 2.1. Preparation of Single Layer PSi

Single-layer PSi thin films were fabricated [28,29] by electrochemically etching *p*-type single-side polished, boron-doped silicon wafers (⟨100⟩, 0.01−0.02 Ω cm, 500−550 μm, Pure Wafer, San Jose, CA, USA) using a 15% *v*/*v* solution of aqueous hydrofluoric acid (HF; 48–51%, Acros Organics, Antwerp, Belgium) in ethanol (Thermo Fisher Scientific, Waltham, MA, USA) in an Advanced Micromachining Tools (AMMT, Frankenthal, Germany) MPSB PSi wafer-etching system. Note that HF is an extremely dangerous chemical and should be handled with the utmost caution [28], and that alternative fabrication methods exist [38]. The wafer was secured in a wafer holder, which was immersed in a HF bath. The wafer holder was subsequently clamped against an inner wall of the etching tool with an o-ring seal, which isolated two half cells, each containing a platinum mesh electrode. The anode was in contact with the exposed back side of the wafer in one of the half cells, the cathode was immersed in the HF bath in the other half cell, and a voltage was applied between the electrodes to provide a constant etching current. Firstly, a sacrificial layer was etched using a current density of 70 mA cm^−2^ for 100 s, which was subsequently dissolved in a 1 M NaOH solution. Secondly, the wafer was washed with deionized (DI) water (resistivity 15 MΩ cm, Elix water purification system, Millipore, Burlington, MA, USA) and ethanol to remove the HF residue and then etched again at a current density of either 55 mA cm^−2^, 40 mA cm^−2^, or 25 mA cm^−2^, to form thin films with different pore size distributions for the different elements in the sensing array. The etching time used to fabricate the PSi films (57 s, 66 s, and 93 s for 55 mA cm^−2^, 40 mA cm^−2^, and 25 mA cm^−2^, respectively) was tailored to give approximately the same thickness, regardless of the etching current density (and associated etch rate). We note that, if experimental conditions such as the HF electrolyte concentration, temperature, and electrode size, position, and conductivity are precisely controlled, then the fabrication is repeatable. Thirdly, the wafer was diced into square 5 mm × 5 mm samples using a DISCO (Tokyo, Japan) DAD3220 dicing saw. Finally, the samples were oxidized at 800 °C in ambient air for 10 min, forming a passivating surface layer of SiO_2_, which is hydrophilic and accumulates a negative surface charge in pH > 2 conditions.

### 2.2. Material Characterization

**Figure 1:**
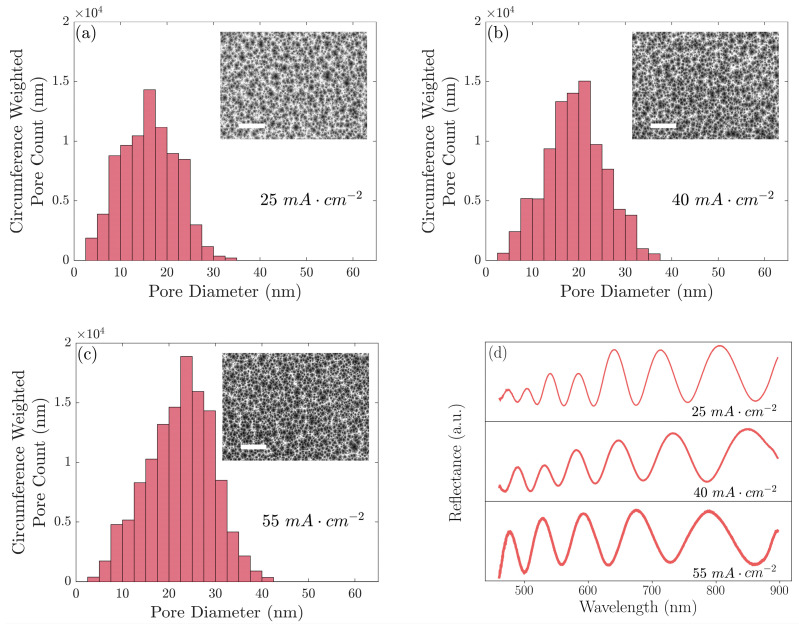
Pore diameter distributions and SEM top-view images for three PSi films, one for each etching current density, formed with current densities of (**a**) 25 mA cm^−2^, (**b**) 40 mA cm^−2^, and (**c**) 55 mA cm^−2^. (**d**) Measured reflectance spectra for each of these PSi films. Scale bars on SEM images are 500 nm.

The properties of the PSi films were measured by analyzing scanning electron microscope (SEM) top-view and cross-sectional images; one PSi sensor for each etching current density was imaged to calculate the pore size distribution (Figure 1) and four PSi sensors for each etching current density were imaged to compute the mean pore size, porosity, and thickness (Table 1). To extract the pore distribution and average pore size, analysis was carried out in MATLAB (R2022b) [39] in a similar manner to that previously reported [40,41]. First, the contrast of the top-view SEM images was made uniform across the image and enhanced using the adapthisteq MATLAB function [42], and then a threshold was used for conversion into a binary image. Isolated pixels were removed and a median filter was applied. The perimeter and area of each of the pores were found using the regionprops MATLAB function, and the pixel to nm conversion was performed using the scale bar in the SEM images. The count of pores in each bin of the pore distribution histogram was weighted by the average perimeter length for each of the pores in that bin. The four top-view SEM images for each etching current density, representing an area of 5.3 μm^2^ or approximately 0.1% of one PSi sensor, were analyzed and the results were averaged to extrapolate the pore size distribution of the PSi as a whole. Similarly, ten measurements across four cross-sectional images were used to calculate the thickness of the PSi layer for each etching current density. For both the pore size distribution and thin film thickness, the errors reported are the standard deviation of the measurements.

### 2.3. Optical Reflectance Measurements

Reflectance spectra were collected by coupling the light from a quartz tungsten light source into a bifurcated optical fiber through one fiber port and measuring the reflected light using an Ocean Optics (Orlando, FL, USA) USB 4000 CCD spectrometer connected to the second fiber port. The height of the fiber was adjusted to form a spot size with a 5 mm diameter on the PSi sensor surface. The output spectra from the spectrometer were fed into a PC running the Ocean Optics Spectra Suite software (version 2), which averaged 100 spectra and saved the result once per second. The reflectance spectra of a single layer of PSi exhibited characteristic Fabry–Perot interference fringes, which are sinusoidal as a function of wavenumber (inverse of wavelength). The sinusoid frequency is equal to the effective optical thickness 2 nL, where n and L are the effective refractive index and thickness of the PSi film, respectively. Molecule adsorption inside the pores causes an increase in the effective refractive index, increasing the frequency of the fringes [28,43].

### 2.4. Experimental Procedure

The PSi samples (5 mm × 5 mm) were washed with water and ethanol and dried under nitrogen. The reflectance spectra of the sensing elements in an array were measured before protein incubation to establish a baseline reference spectrum; representative reflectance spectra for each etching current density are shown in Figure 1d. To keep the experimental conditions as consistent as possible across the large number of experiments needed to compile a reasonable dataset for analysis, bulk protein solutions were prepared in DI water (10 g/L) and stored at 4 °C. The bulk protein solutions were further diluted with either pH 10 and pH 4 reference standard buffers (Sigma-Aldrich, Burlington, MA, USA) and water to prepare three concentrations of each protein (2 g/L, 0.2 g/L, and 0.02 g/L). The ratio of DI water:reference standard buffer (pH 4 or pH 10) was maintained at 1:4 for all the experiments; this ratio was chosen as a tradeoff between maximizing the proportion of the buffer for optimal pH control and stability and a sufficiently high volume of protein solution to enable a large dynamic range of accessible concentrations using 10 g/L stock protein solution. The pHs of the solutions using the pH 4 buffer and pH 10 buffer were found to be 4.0 ± 0.1 and 10.0 ± 0.1, respectively, measured using a Mettler Toledo (Columbus, OH, USA) Seven Easy pH meter. The corresponding molar concentrations of the protein solutions were 30 μM, 3 μM, and 300 nM for BSA (pI = 4.63, MW = 66.4 kDa, Thermo Fisher Scientific), 45 μM, 4.5 μM, and 450 nM for OVA (pI = 4.54, MW = 44.3 kDa, Thermo Fisher Scientific), and 30 μM, 3 μM, and 300 nM for avidin (pI = 10, MW = 66–67 kDa, Thermo Fisher Scientific). A 20 μL volume of protein solution was drop cast on each sensing element in the array and left to incubate for 2 h under ambient conditions in a sealed container to prevent evaporation. Afterwards, the sensors were placed in an 800 mL water bath for 10 s, and then removed and dried under nitrogen. The purpose of this wash was to remove unbound molecules from the PSi surface and inside the pores. A much smaller number of weakly bound molecules would also be removed, but the reflectance change during the washing was small and almost entirely independent of the wash duration, indicating that most molecules were adsorbed strongly enough to remain in the pores. Each sensing element in the array was then dried under nitrogen and measured again, and the resulting spectrum was compared to the reference spectrum before protein solution exposure by calculating the Morlet wavelet phase response [43]. The total number of elements in each array that were exposed to one concentration of a given protein was twelve, corresponding to three average pore sizes, two buffers, and two repeats. Since each sensor element was 5 mm × 5 mm, the total area of each sensor array was 300 mm^2^. An overview of the PSi sensor array is shown in Figure S1.

### 2.5. Data Analysis

Linear discriminant analysis (LDA) was used to reduce the dimensionality and visualize the six-dimensional sensor array responses in 3D, elucidating the separability of the three proteins. LDA is a statistical method that determines a series of linear projections of a given training dataset that best separate data points by their associated class [44]. This is achieved by maximizing the ratio of between-class variance to within-class variance.

Support vector machines (SVMs) are supervised optimal margin classifiers, chosen due to their stability, interpretability, and applicability to small datasets [45].

## 3. Results and Discussion

The PSi thin films were fabricated using three different current densities, achieving three distinct pore size distributions with different average pore sizes. The pore size distributions were weighted by the circumference of the pores, which is proportional to the number of binding sites.

Table 1 shows the average pore diameter and thickness, which were determined by analyzing the SEM images, porosity, which was calculated from the optical reflectance measurements, and average effective optical thickness and refractive index, which were calculated through an analysis of the reflectance spectra, as a function of the etching current density.

The appropriate selection of PSi formation conditions, including current density, etching time, HF concentration, and silicon wafer doping, enables a relatively wide range of tunability in the PSi properties shown in Table 1 [28]. Both Figure 1 and Table 1 show the pore size distributions shifting to larger diameters as the etching current density was increased. Important for size selectivity, the fraction of larger (>30 nm) pores dramatically increased by an order of magnitude as the etching current density increased from 25 mA cm^−2^ to 40 mA cm^−2^ and increased again by a factor of nearly three as the etching current density was increased to 55 mA cm^−2^. We believe this metric was a dominant effect governing the response of the PSi films to protein exposure compared to the average pore size, which exhibited only a modest increase with etching current density. While the porosity also increased as a function of the etching current density, porosity changes would alter the response of all the proteins proportionally and, consequently, would provide no additional discriminatory information, unlike the pore size, which had an important differential effect [46]. We note that our analysis of the top-view SEM images established a clear general trend of an increasing pore size with an increasing etching current density. Thresholding to convert the images from greyscale into binary was performed manually and the fine structure of the pore branches within the pores was not rigorously taken into account. Consequently, although the standard deviation of the repeated independent measurements was low, there could have been systematic uncertainty associated with the resolution of the SEM images and manually chosen thresholds. Further detailed analyses of the pore size and morphology for PSi layers fabricated using a range of different etching conditions can be found elsewhere [30,47,48].

Solutions of BSA, OVA, and avidin at three different concentrations and a negative control with no protein were prepared in DI water and either pH 4 or pH 10 buffers at a ratio of 1:4, resulting in overall solvent pHs of 4.0 ± 0.1 and 10.0 ± 0.1, respectively. The solutions were drop cast and incubated for 2 h on the PSi films with three different pore sizes, resulting in six combinations of pore sizes and pHs in the sensing array. Sixteen of these sensor arrays were constructed for every concentration of each protein, and randomly sampled pairs of array measurements were averaged to reduce the variance in the response arising from the nature of the adsorption phenomenon, yielding eight independent repeats, allowing for an estimation of the mean and variance for every experimental condition. The reflectance spectrum of each sensor array element was measured before and after the protein solution exposure, and spectral shifts indicative of infiltration and adsorption in the pores were transduced by processing the spectra using Morlet wavelet phase analysis [43]. Here, infiltration refers to the diffusion of molecules into the pores and adsorption refers to the attachment of the molecules to the pore walls.

**Figure 2:**
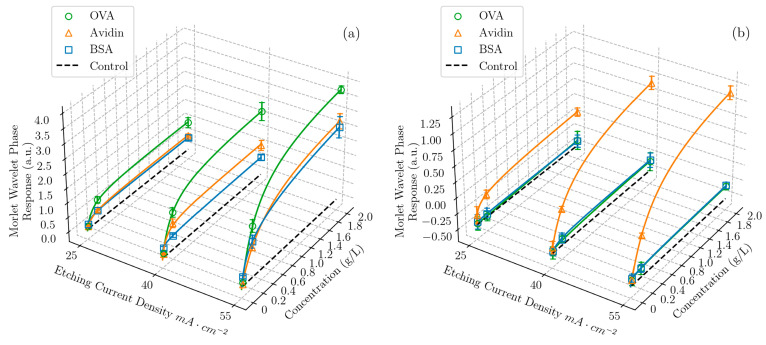
Morlet wavelet phase response as a function of both etching current density (proportional to average pore size) and concentration for three proteins—OVA, BSA, and avidin—and a negative control with no protein, in solutions of DI water and (**a**) pH 4 and (**b**) pH 10 buffer, in a ratio of 1:4 (*v*/*v*). The data points represent the average value of sixteen measurements taken at the same condition and the error bars represent the standard deviation of the measurements. Each response curve was fit with the Redlich–Peterson adsorption isotherm [50]. Equivalent 2D plots are shown in the Supporting Information (Figure S2).

From the response of each sensor in the array (Figure 2), it was clear that the differences between the proteins were subtle at low concentrations, but easily discriminable by eye at the highest concentrations, allowing for several observations to be made. Firstly, the response to all the protein solutions was proportional to the pore size: as the pore size distribution shifted to higher diameters and the average pore size increased, the response increased, as expected [49]. Notably, the relationship between the response and pore size for a given protein was not linear, and there were three regimes to consider: (1) at higher average pore sizes, the proteins experienced essentially uninhibited entry and diffusion into the majority of the pores, (2) at lower average pore sizes, inhibited molecular transport began to pinch off the response because there were few pores large enough to permit infiltration and adsorption, and (3) at intermediate pore sizes, there was a transition between the other two regimes.

A second observation can be made regarding the effect of pH on the response of the PSi films. Multiple studies have shown that the maximum infiltration of proteins in the pores occurs when the pH environment is at the isoelectric point of the protein, resulting in a net neutral molecular charge [51,52]. This condition provides the minimum inhibition of protein transport and promotes close packing in the pores by avoiding extensive protein–protein and protein–PSi interactions. Accordingly, the results in Figure 2 show that the largest response of the PSi to each of the proteins occurred when the pH of the solution was approximately equal to the isoelectric point of that given protein. We note that the surface of the oxidized PSi was negatively charged when the pH of the environment was above 2, which was the case for all the experiments carried out in this work [53]. We further note that, while the pH at which maximum adsorption occurred was indicated simply by a molecule’s isoelectric point, the dependence of the adsorption characteristics on the pH will generally have a different shape for every molecule, providing another fingerprinting mechanism: the properties governing infiltration and adsorption, such as protein charge distribution [54], agglomeration, and conformational changes [55], are unique to any given molecule and are pH-dependent.

Thirdly, by combining the first two observations, we can understand that, when the pH 4 buffer was used, close to the isoelectric point of both OVA and BSA, a higher response was given by OVA due to its smaller molecular size.

Finally, for the solutions using the pH 10 buffer, the baseline was negative due to the oxidation and dissolution of the PSi matrix by the hydroxide ions present in the basic protein solutions [28,56], which competed with the rising response due to the protein adsorption in the pores. This dissolution effect diminished the sensor array response to all three proteins and notably caused the sensor array response to avidin in the pH 10 buffer to be lower than that of avidin in the pH 4 buffer, even though the latter condition was farther from avidin‘s pI. Importantly, machine learning models can utilize all this information to discriminate between proteins, implicitly taking the complex interplay of adsorption and dissolution effects into account.

To summarize, each protein at each different concentration gave a unique combination of responses to each of the sensor elements in the sensor array, which all had different properties. The resulting fingerprint of the responses for this protein was distinct from that of other proteins. For example, while 2 g/L of BSA gave a very similar response to 2 g/L of avidin when exposed to a sensor etched with 55 mA cm^−2^ in the pH 4 buffer, when the pH 10 buffer was used instead, the BSA response was negligible and the two proteins were easily distinguishable.

**Figure 3:**
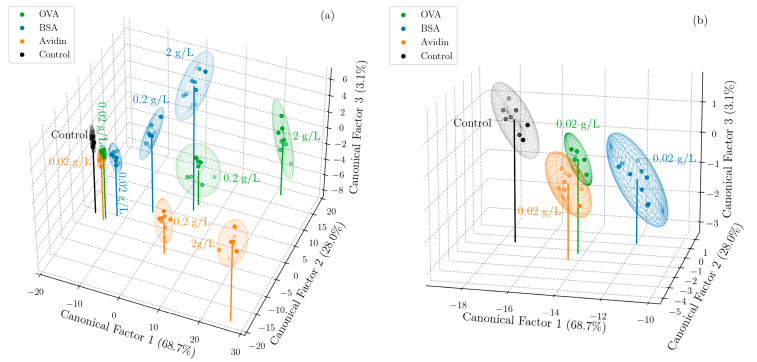
Canonical score plot of the three dominant factors obtained from LDA for (**a**) 3 proteins (OVA, BSA, and avidin) at 3 concentrations (2 g/L, 0.2 g/L, and 0.02 g/L) and a negative control with no protein, and (**b**) the same 3 proteins at the lowest concentration (0.02 g/L) and a negative control. The ease of classification and quantification of the proteins at the higher concentrations can be observed, as well as the separability at a low concentration. See Video S1 in Supporting Information for additional views of the data in (**a**).

Following the optical measurements and Morlet wavelet phase analysis, LDA was used to reduce the dimensionality of the sensor array response matrices from six-dimensional (due to the combination of the three unique formation conditions of the PSi films and two pH values used in the experiments) to three-dimensional, enabling a visualization of the degree to which the three proteins could be separated. LDA was selected for its ability to maximize the ratio of between-class variance to within-class variance [44]. Figure 3 shows the PSi sensing array response to different concentrations of the target proteins, projected along the dominant three canonical factors given by LDA with 95% confidence ellipsoids overlaid and stems indicating the class means; a rotating graphic is included in the Supplementary Materials.

We note that an additional larger pore size (obtained using an etching current density of 70 mA cm^−2^) was included in the preliminary experiments but was found to provide negligible additional discriminatory value due to its high correlation with responses to neighboring pore size features, namely PSi films fabricated using a current density of 55 mA cm^−2^.

Next, the concentrations of each of the three proteins were classified with support vector machines (SVMs) using a linear kernel and regularization hyperparameter C = 100 (informed by the analysis of a small preliminary dataset). A SVM model was trained on the reduced dimensionality dataset given by LDA, which improved the accuracy by reducing noise. The model, when coupled with leave-one-out cross validation, gave accurate predictions of both the protein type and concentration in 100% of the cases. To test the ability of the model to classify unseen concentrations, the same SVM model with a linear kernel was retrained to classify the protein type only, and a test set was compiled by carrying out further experiments, in which sixteen sensor arrays were exposed to OVA at a new concentration of 0.1 g/L. By averaging two measurements, these further experiments yielded eight new data points. This choice of protein and concentration was made to rigorously test the system. It was clear that avidin could be trivially classified due to the large differential effect of pH, whereas in the training dataset (Figure 3), OVA and BSA were less easily discriminable, particularly at low concentrations. The independent test set was classified with an 87.5% accuracy (one protein was misclassified of the eight in the test set), illustrating the promise of this approach for classifying proteins of an unknown concentration. We note that, to avoid data leakage, the mean and standard deviation of the training data set were used to standardize all the data of both the test and training sets. For the same reason, the canonical factors given by LDA when applied solely to the training set were used to transform the test set. The same process was followed in the case of leave-one-out cross validation, for which the data point left out of the training set for classification was considered the test set. The accuracy of the classification using these SVM models, summarized in Table 2, could be increased with a larger array incorporating more pore sizes and pH values to give more discriminating power. Additionally, because no cross validation for the model selection or hyperparameter tuning was carried out due to the limited amount of data, the accuracies reported here are a lower bound of what could be achieved with more data and a more complex optimized model. We note that, while the third canonical factor in Figure 3 represented a small percentage of the discriminatory power (3.1%), it played a critical role in separating BSA and OVA, which have a similar pI but different molecular weights, and almost identical trajectories when projected only onto the first two canonical factors. Consequently, the third canonical factor has a large contribution to the accuracy of models trained on the dimensionality reduced training set. As a result, the discriminatory power was not the best indicator of feature importance in the context of discriminating proteins, which was partly a consequence of investigating different concentrations of each target molecule.

To investigate the capability of this new approach to sensing without capture agents in more complex sensing scenarios, including detecting target molecules in biologically relevant media, future work will explore increasing the size of the sensing array to encompass additional degrees of freedom (e.g., including the ionic strength of analyte solution, surface charge and hydrophobicity, and the real-time optical monitoring of adsorption and diffusion). It is anticipated that this larger array would be able to distinguish between a larger number of proteins and other species of interest. Given that the average pore size of PSi is tunable in the range from <2 nm to >100 nm [28], the sensing array is potentially applicable to a wide range of molecules with a size of ≤100 nm. Moreover, the detection limits of the sensing array can be improved simply by reducing the size of the individual PSi sensor elements while maintaining the same volume of solution, increasing the number of molecules per unit area, and, consequently, the magnitude of the spectral shift. Finally, we note that a scalable and cost-effective smartphone-based imaging approach to measuring the optical signals [57] could be implemented, enabling the response of an almost arbitrarily large array of PSi sensors to be captured as a function of time.

## 4. Conclusions

We report the first demonstration of a new approach to molecular identification and quantification based on an array of porous silicon sensors, each with a unique combination of properties but no functionalization or capture agents. This system was able to classify and quantify three proteins separately with a similar molecular size down to concentrations of ~300 nM using optical reflectance measurements and machine learning analysis. An accuracy of 100% was achieved for the proteins and concentrations previously encountered in the training set, and a previously unseen independent test set collected using an intermediate concentration of one of the proteins was classified with 87.5% accuracy. The design of this system could obviate the need for capture agents, paving the way for cheaper, more robust, and quicker-to-develop sensors that provide medical diagnostics, environmental monitoring, and food safety systems to resource-limited environments.

## Figures and Tables

**Table 1:** Average pore size, porosity, thickness, and fraction of pores larger than 30 nm determined from measurements of four PSi thin films for each of the three etching current densities used to fabricate elements of the sensor array.

Etching Current Density	25 mA cm^−2^	40 mA cm^−2^	55 mA cm^−2^
Mean Pore Diameter (nm)	12.0 ± 0.2	15.1 ± 0.2	17.3 ± 0.2
Pore Diameter Standard Deviation (nm)	6.0 ± 0.2	6.7 ± 0.3	7.9 ± 0.3
Thickness (μm)	1.78 ± 0.01	1.94 ± 0.01	2.04 ± 0.01
Mean Effective Optical Thickness in Air (μm)	6.91	6.34	5.82
Mean Effective Refractive Index in Air	1.94 ± 0.02	1.63 ± 0.01	1.43 ± 0.01
% Porosity	53 ± 1	61 ± 1	66 ± 1
Fraction of Pores > 30 nm	0.2 ± 0.1%	2.0 ± 0.4%	5.8 ± 1.3%

**Table 2:** Summary of performance of SVM classifier with a linear kernel.

Test Procedure	Prediction Target	Accuracy
Leave-one-out cross validation (previously seen concentrations)	Protein type and concentration	100%
Independent Test Set (previously unseen concentration)	Protein type	87.5%

## Data Availability

Data are available upon reasonable request from the corresponding authors.

## Supplementary Materials

The following supporting information can be downloaded at: https://www.mdpi.com/article/10.3390/bios13090879/s1, Video S1: Rotating 3D view of LDA canonical score plot to visualize protein separability, Figure S1: Schematic diagram overview of the PSi capture agent free sensing system, showing the sensor array with two independent duplicates, protein solution being drop cast and reflectance measurements being carried out, Figure S2: An alternative 2D plot visualization of Figure 2, showing a side-by-side comparison of Morlet wavelet phase response curves for each etching current density and concentration of the three proteins (OVA, BSA and avidin), and Table S1: Weightings of the original 6 dimensions corresponding to the six unique experimental conditions used to project the original high dimensional data onto each canonical factor.
