# Supplementary material for: Protein Identification and Quantification Using Porous Silicon Arrays, Optical Measurements, and Machine Learning
Source: Biosensors (Basel). 2023 Sep 9;13(9):879. doi: 10.3390/bios13090879 (PMC10526835; doi:10.3390/bios13090879)
Supplement: Supplementary file 1 [file biosensors-13-00879-s001.zip › biosensors-2581568-supplementary.pdf]

Supporting Information

Protein Identification and Quantification using Porous Silicon Arrays, Optical Measurements, and Machine Learning

Simon Ward [1], Tengfei Cao [2], Xiang Zhou [3], Catie Chang [1], and Sharon Weiss [1,2,*]

1 Department of Electrical and Computer Engineering, Vanderbilt University, Nashville, Tennessee 37235, USA
2 Interdisciplinary Material Science Program, Vanderbilt University, Nashville, Tennessee 37235, USA
3 Department of Chemistry, Vanderbilt University, Nashville, Tennessee 37235, USA
* Correspondence: sharon.m.weiss@vanderbilt.edu

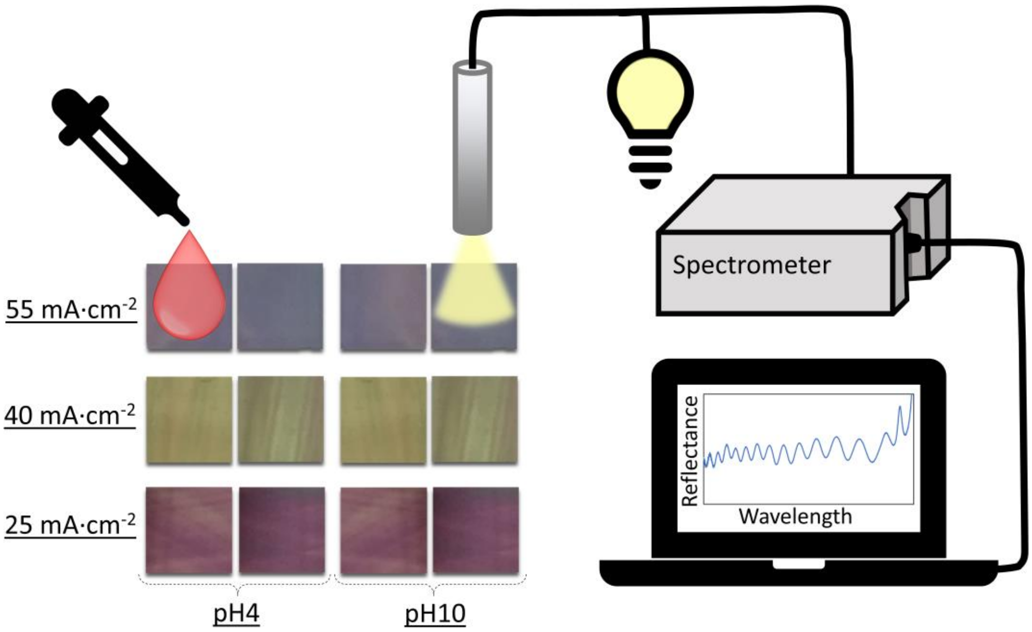

Figure S1. Schematic diagram overview of the PSi capture agent free sensing system, showing the sensor array with two independent duplicates, protein solution being drop cast and reflectance measurements being carried out.

Table S1. Weightings of the original 6 dimensions corresponding to the six unique experimental conditions used to project the original high dimensional data onto each canonical factor.

	**Canonical Factor 1 (68.7%)**	**Canonical Factor 2 (28.0%)**	**Canonical factor 3 (3.1%)**
55 mA cm^{-2}, pH 4	0.76	0.63	0.70
55 mA cm^{-2}, pH 10	0.43	-0.88	-0.08
40 mA cm^{-2}, pH 4	0.39	0.17	-0.54
40 mA cm^{-2}, pH 10	0.25	-0.32	0.01
25 mA cm^{-2}, pH 4	0.18	0.18	-2.15
25 mA cm^{-2}, pH 10	0.05	-0.07	-0.57

Table S1 shows the weightings of each of the features of the original 6D response matrices which correspond to each canonical factor. For example, the response of the sensor element etched using 55 mA cm^{-2} and exposed to protein solution in pH 4 buffer is the highest contributing feature to the first canonical factor, whereas the sensor element etched with 25 mA cm^{-2} and using pH 10 buffer has almost negligible contribution to that canonical factor. These weightings can be interpreted with reference to the LDA score plot (Figure 3). From Figure 3a we can observe that this first canonical factor predominantly separates out different concentrations of the proteins rather than separating out the different types of proteins themselves. Returning our attention to Table S1, we can then understand why the largest weighting in the first canonical factor is the feature representing the largest average pore size using pH 4 buffer. These are the conditions that maximize response across all proteins, by enhancing molecular transport based on molecular weight and isoelectric point, and therefore give the largest differential between high and low concentrations. Visually, from Figure 3a, the second canonical factor separates avidin from the other two proteins and hence for this canonical factor, the weighting with the largest magnitude is the largest average pore size using pH 10 buffer, generating the largest differential between different isoelectric points. Furthermore, the weightings have opposite signs for conditions using pH 4 and pH 10 buffers. Consequently, the projection of avidin onto canonical factor 2 will be negative, given that it has a much higher relative response in pH 10 buffer, whereas the projection of BSA and OVA onto canonical factor 2 will be positive due to their relatively high response in pH 4 buffer conditions. The third canonical factor primarily separates molecules by molecular weight according to Figure 3a. The sign of the weighting enhances the differential response since the higher molecular weight molecules (BSA and avidin) have a relatively high response to the higher average pore size sensors, but a much lower relative response at lower average pore size sensors for which the weighting is negative. On the other hand, OVA has a much higher relative response at the lower average pore size sensors as well as the high pore size. Consequently, BSA and avidin responses will mostly lie above zero in the 3rd canonical factor axis, whereas the response to OVA will largely reside below zero. This analysis suggests that in our model system test, the LDA results are consistent with physical intuition. However, one important caveat to this analysis is that there may be other molecular properties that are also correlated with pore size or buffer pH, other than molecular weight and isoelectric point, and which play a significant role in the discrimination of the three proteins. A key advantage of utilizing machine learning is that these correlations need not be known to be leveraged: complex relationships can be learned in a data driven approach to discriminate molecules that may be almost impossible to predict.

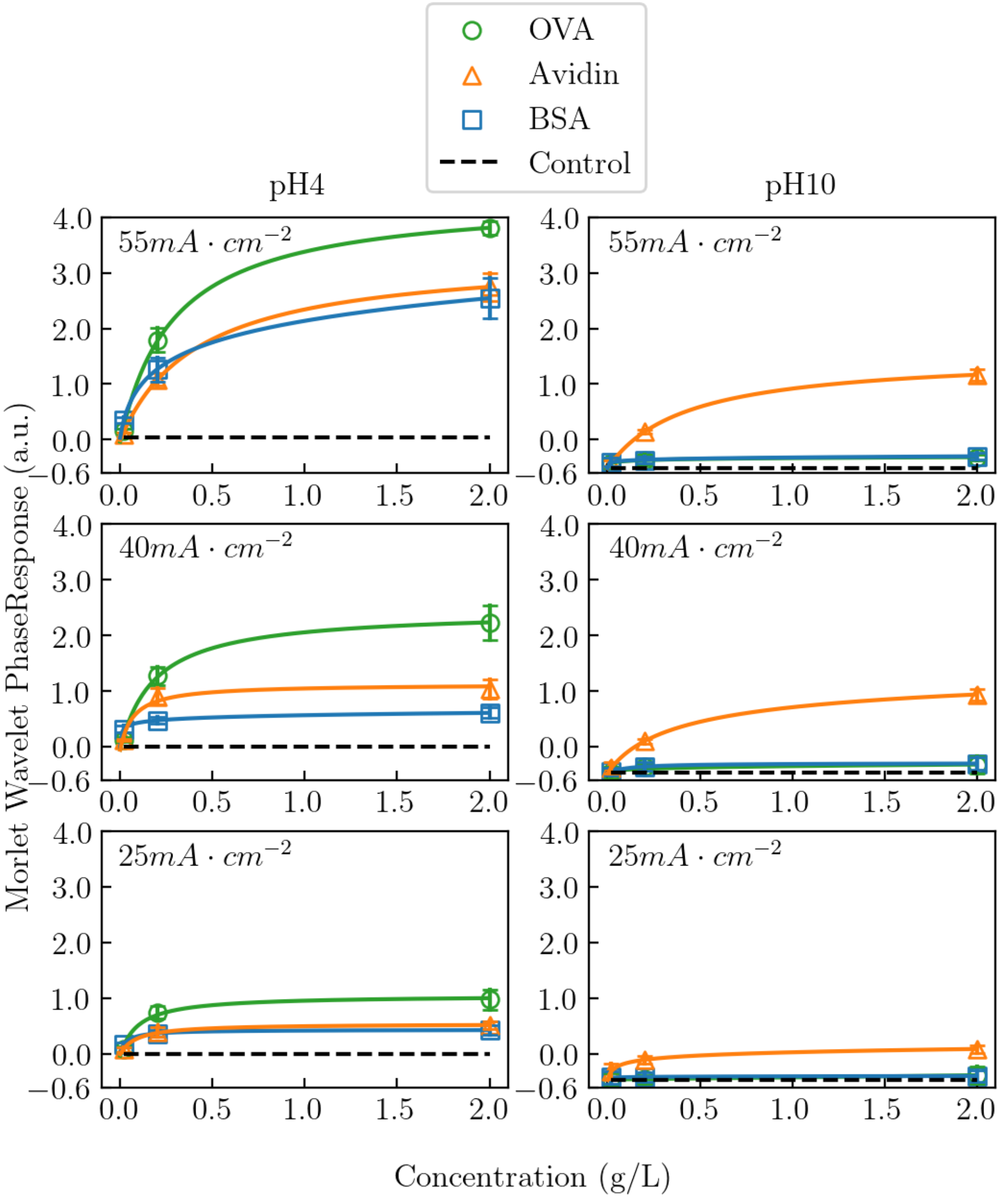

Figure S2. An alternative 2D plot visualization of Figure 2, showing a side-by-side comparison of Morlet wavelet phase response curves for each etching current density and concentration of the three proteins (OVA, BSA and avidin).
